# A qualitative study exploring the perceptions and understandings of advance care planning by people with treatable but not curable cancer

**DOI:** 10.1177/02692163251363752

**Published:** 2025-08-31

**Authors:** Ruth Board, Sean Hughes, Katherine Stewart, Tomoko Lewis, Sheila Payne

**Affiliations:** 1 Lancashire Teaching Hospitals NHS Foundation Trust, Lancashire, UK; 2 International Observatory on End of Life Care, Health Innovation One, Lancaster University, Lancaster, UK

**Keywords:** Advance care planning, cancer, palliative care, communication, qualitative research

## Abstract

**Background:**

Advance care planning for those with treatable but not curable cancer is considered good practice because innovations in treatment options make prognostication much more uncertain. Little is known about how such patients approach future planning.

**Aim:**

To elicit the perceptions and understandings of advance care planning by patients with treatable but not curable cancer.

**Design:**

Qualitative, in-depth interviews with patients were analysed using a reflexive thematic approach within a social constructivist paradigm.

**Setting/participants:**

Twenty patients with treatable but not curable cancer were recruited to the study from a cancer care centre. Nine patients choose to be accompanied by family members.

**Results:**

Four integrated themes highlighted that increasing availability of on-going and novel treatments, with survival beyond initial prognosis, impeded advance care planning and contributed to a sense of uncertainty. Participants described the existential difficulty of holding contradictory thoughts about living with cancer while simultaneously contemplating end of life preferences. Most participants did not recognise conversations with clinicians as advance care planning, including ‘do not resuscitate’ decisions. Most participants preferred to discuss future care, social and funeral arrangements with family. A few, with caring responsibilities, proactively undertook advance care planning.

**Conclusions:**

This study highlights challenges in advance care planning for those with treatable but not curable cancer, especially when uncertain about disease progression. Data suggest that a separation between conversations about medical planning and that of a more social and personal nature may be needed. Further research should investigate the impact of uncertainty of survival on advance care planning practice.

**What is already known about this topic**

- Advance care planning is considered good practice in palliative and end of life care and is promoted in health policy.
- There is no standardised approach to advance care planning in practice.
- There is recent debate about the utility and effectiveness of advance care planning in palliative care contexts.

**What this paper adds?**

- Most patients did not recognise the concept of advance care planning and did not welcome conversations with health care providers about future planning despite many participants having done this prior to interview.
- Patients with treatable but not curable cancer live with uncertainty of prognosis in the context of ongoing and new treatment options, making advance care planning problematic.
- Most patients preferred to discuss future care, social and funeral arrangements within families, if at all.

**Implications for practice, theory or policy**

- The principles of future care planning can be introduced early in treatment without making them specifically about planning for the last days of life.
- Healthcare professionals in cancer and palliative care may need to ensure that future care planning discussions evolve over time, with decisions made being routinely revisited in light of changes in disease progression, treatment options and prognosis.
- Future policy guidance on advance care planning needs to take account of the changing treatment landscape for those with treatable but not curable cancer.

## Background

Advance care planning is internationally recognised as a component of good palliative care which is well established in countries with developed palliative care services.^1^ Bibliometric analysis demonstrates an exponential rise in advance care planning research.^2^ While advance care planning has demonstrated benefits in some contexts, there remain concerns about its complexity, utility and limited adoption.^3,4^

We use the European Association for Palliative Care international consensus definition of advance care planning which enables ‘individuals to define goals and preferences for future medical treatment and care, to discuss these goals and preferences with family and health-care providers, and to record and review these preferences if appropriate’.^5^ It comprises a voluntary process involving iterative conversations which seeks to elicit and record the person’s wishes and preferences for care in case they lose mental capacity.

An international umbrella review indicates that advance care planning improves patient navigation in cancer care.^6^ A study in the Netherlands demonstrated that it has the potential to improve communication between patients and healthcare providers, enhance the quality of life and well-being of patients and their family members, reduce aggressive treatments and unnecessary hospitalisations and result in better concordance between preferred and actual place of death.^7^ Systematic reviews have shown it to have a positive impact on patients’ experience of cancer care including an increased awareness of prognosis and improved decision making around end-of-life options.^1,8–10^ However, a large cluster randomised control trial in patients with advanced cancer in six European countries, showed no effect on quality of life.^11^ A qualitative analysis of advance conversations from this trial indicated that fundamental concepts underlying these conversations did not resonate with patients.^10,12^ A recent meta-review of advance care planning interventions identified 39 reviews in the last decade but highlighted heterogeneity of methods.^13^

Advance care planning assumes a clear understanding of prognosis and a willingness to contemplate the end of life.^5^ The recent emergence of innovative treatments for cancer, often combined with concurrent co-morbidities, mean that people may face uncertain futures due to unpredictable illness trajectories.^14^ There are increasing sub-cohorts of patients with treatable but not curable cancer, for whom prognostication is challenging.^15^ While clinical uncertainty is pervasive in many conditions, the lived experience of managing this uncertainty and how it is appraised by patients with treatable but not curable cancer and the influence it may have on future planning is unexplored.

Most published evidence on advance care planning is not specific to patients with treatable but not curable cancer, yet there are likely to be more in this sub-group as treatment options increase, even for those with advanced disease. Our study aimed to explore this gap in knowledge and to elicit the perceptions and understandings of advance care planning from these patients as this may alter the approach to supporting these patients over a longer period of time by healthcare professionals in cancer and palliative care.

## Methods

### Design

Our research was conducted within an interpretivist paradigm in which we understand reality to be subjective, multiple and socially constructed. We employed semi-structured interviews to gather data and adopted a thematic analytical approach with a reflexive orientation based on the evolving methods of Braun and Clarke.^16,17^

### Ethics and reporting

Ethical approval was received from NHS Research Ethics Committee ref: 23/SC/0090, 19.4.2023. Funding was provided by the Rosemere Cancer Foundation (UK Registered Charity Number 1131583). Consolidated criteria for reporting qualitative research (COREC) guidelines have been followed in reporting the findings.^18^

### Participants and recruitment

Patients with treatable but not curable cancer receiving palliative systemic therapies in a cancer care centre were purposively identified by treating clinicians in oncology and Palliative and Supportive care clinics as meeting the study inclusion criteria (Table 1). Patients could choose a family member to accompany them for support during the interview, if desired.

**Table 1.** Inclusion criteria for patients.

Inclusion
Patients receiving systemic treatment for cancer with palliative intent that is, treatable not curable
Patients with the cognitive ability to engage in conversation and provide consent
Aged 18 years or over
Clinician discretion was used when determining which patients would be suitable for inclusion to minimise patient distress.

Eligible patients were approached by a clinician to determine their interest, and if so, were provided with a participant information sheet, an expression of interest notification, a consent form and a return envelope. If the patient wished, similar packs for accompanying family members were distributed. On receipt of the expression of interest form, the researcher contacted potential participants by telephone to answer any questions, and to determine if they wished to proceed following at least 24 h to consider their decision. Once verbal consent to continue was established, the researcher made an appointment to conduct the interview. Interviews were carried out in a place preferred by the participant, normally the person’s home, the local hospital and, in one case, the university. Twenty patients were recruited to the study with nine choosing to be accompanied by family members. Five people who expressed an initial interest did not complete an interview due to lack of contact, changed their mind and one was too ill. One hundred patient packs were distributed to clinicians, it is therefore estimated that 75 packs were unused. Multiple packs were distributed to ensure multiple clinicians in the oncology department had access to the study paperwork ensuring a broad and representative cohort for participation.

### Data collection

Semi-structured interviews were conducted by SH, an experienced qualitative researcher, formerly a palliative care social worker, qualified in humanistic integrative counselling who had no prior relationship with participants. The topic guide (Supplemental Material File 1) was developed by the authors (SH, RB and SP) based on a scoping of the literature and clinical experience, and pilot tested with three people with experience of cancer. Written and verbal consent was obtained from all participants. Demographic questions including gender, ethnicity and faith/religion were asked prior to the interview. Interviews were digitally audio recorded and uploaded into a secure digital space, before deletion. The topic guide was used to facilitate the conversation, but participants were encouraged to explore aspects of advance care planning that held resonance for them. A distress protocol was developed should participants become upset during the interview, which occurred twice. Contemporaneous field notes were recorded after each interview and used in a reflexive journal to capture feelings and ‘hunches’ and contributed to the analysis. Interviews were conducted between April and October 2024. Repeat interviews were not conducted, nor were interview transcripts or findings shared with participants, some of whom were seriously ill and eight subsequently died within the following 5 months.

### Data analysis

**Figure 1.**
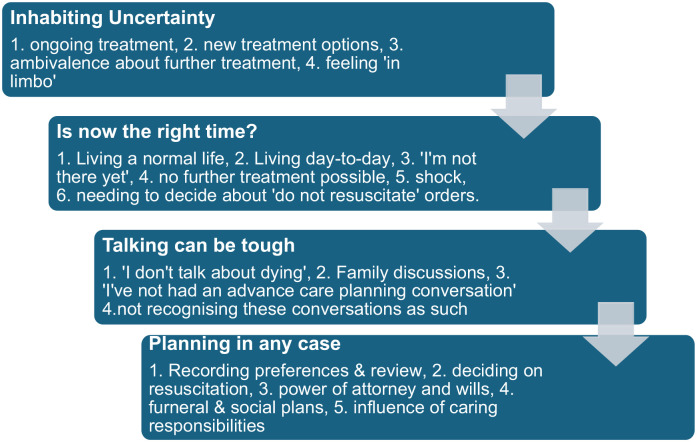
The processes that influence engaging with advance cancer planning for patients with treatable but not curable cancer, based on four themes and subthemes.

Interviews were transcribed verbatim and pseudo-anonymised (SH). Transcriptions were uploaded into NVivo qualitative analysis software for storage and retrieval during the analytic process (Supplemental Material File 2: coding tree). Familiarisation with the data began immediately through repeated reading and reflection on the transcripts. SP and SH independently began a process of generating initial codes inductively, with frequent meetings to collaboratively develop themes. Themes were reviewed, named and a graphic schema constructed to illustrate their relationship to sub themes and to each other (Figure 1). At this point, a meeting with the clinical team (RB, KS and TL) who had recruited participants, was convened to discuss initial findings. In discussion with the clinical team, new insights were gained – particularly in relation to the language used in advance care planning conversations between clinicians and patients – which guided further coding of the data set (SH and SP) and a refinement of themes. During analysis, frequent reference to the researcher’s reflexive journal was made in order that contextual and situational factors were not lost in the process and to ground the analysis in participant experience.

## Findings

We recruited a purposive sample to establish information power, a conceptual model which contends that the larger the amount of relevant data the sample holds, the lower the number of participants needed.^19^ We interviewed 20 patient participants (Table 2), 9 of whom choose to be accompanied by family members including spouses and adult children.

**Table 2.** Characteristics of patient participants.

Characteristics and total numbers	
Gender	
Female	12
Male	8
Age: (range: 47-87, median: 70)	
40–49	1
50–59	2
60–69	7
70–79	8
80–89	2
Ethnicity	
White British	20
Primary cancer site	
Lung	7
Gynaecological	4
Gastro-intestinal	4
Melanoma	3
Bladder	1
Tonsil	1
Faith/religion	
Christian	15
None	5
Interview location	
Home	17
Hospital	2
University	1

Interview duration: median 25.5 min (range 11–45 min).

Eight participants died within 5 months of interview.

We identified the processes, not necessarily linear, that patients with treatable but not curable cancer described in their engagement with advance care planning, based on four integrated themes and sub themes (Figure 1). Transitions between processes may be precipitated by changes in disease progression or personal and social circumstances, although not everyone progressed through all elements. These are described below with illustrative quotes with anonymised names but genders unaltered.

### Inhabiting uncertainty

Participants referred to a variety of concerns about uncertainty for the future which in some cases appeared to block attempts at planning for, or even contemplating, what might lie ahead. While they understood that their cancer was incurable, they recounted numerous instances of further treatment which extended survival. For example, Pamela’s clear expression of the paralysing effect of ‘not knowing’ what the future held was replicated in other accounts where patients were offered a range of treatment options but were ambivalent about accepting them. For some (like Betty), this resulted in surviving long after their anticipated life expectancy when initially diagnosed.

*I don’t know what’s gonna happen . . . I don’t know when it’s gonna happen . . . I don’t know how it’s gonna happen . . . I don’t know where it’s gonna happen . . .* **Pamela**

*They keep saying like: ‘this is your last chance, like your last treatment’ and then when you go back it’s* ***another*** [her emphasis] *treatment . . . it’s a bit weird.* **Betty**

Not knowing how their illness might progress and what it would mean in terms of wishes and preferences inhibited forward planning or justified not planning, at least, not at this point.

*Well, I suppose it depends . . . so it depends what sort of condition you’re in, doesn’t it? if you’re just gradually going downhill and then it shuts off . . . but if you’re in absolute excruciating pain, then the planning for, you know, for the end is going to be different, isn’t it?* **Tom**

Participants’ accounts included a wide range of different experiences of living with uncertainty and their observations on how it impeded the ability to make decisions or plans. There were perceptions of experiential angst or being ‘in limbo’ which some dealt with by taking a day-to-day approach and avoiding thinking ahead or engaging in planning conversations when these were raised (as described by Mary). An alternative strategy was to refer to stories of people they knew or had heard of who had survived cancer, or to cite information gleaned from the media or online as a way of offsetting uncertainty and remaining hopeful that something similar could happen for them. This helped to mitigate distress and enabled a focus on positive experiences.

*I went to see the palliative care nurse . . . I don’t want to know how long I’ve got to live. So, I’ve told them that. So, it was just a nice chat about how I was feeling, erm . . . how I was taking it day by day, which I am. And really that, that was all . . . there was no further plans ‘cause I don’t want to know’.* ***M*ary**

### Is now the right time?

We identified that participants thought that the timing of advance care planning conversations would be triggered by receiving an explicit ‘palliative’ diagnosis or informed that no further treatments were available. Their understanding of palliative care was that it was delivered only when dying was imminent. Thus, there appeared to be a tension between believing that they were not yet ‘terminally’ ill and yet being invited to contemplate that possibility. There was a focus on (normal) daily living rather than the future.

*But I don’t know when is the best time* [for an advance care planning conversation] *when they put you on the palliative care pathway? is that the time really that you should start um talking about . . . rather than . . . cause I’m not on that yet, so am I anticipating? it’s a difficult one to know, isn’t it? Maybe when you go on to the palliative . . . when they turn around and say: ‘That’s it, Sophie, there’s not much more we can do’, maybe that’s the time to start looking at it . . .* **Sophie**

*Personally, I think when you get the terminal prognosis someone needs to say to*

*you: ‘here’s a little leaflet for you to think about things later on’ and then when*

*you’re told: ‘there’s no more treatment we can do for you now other than make you comfortable’ that’s when things need to kick up a gear.* **Peter**

As the preceding extracts illustrate, participants often wished for advance care planning to be offered when they felt ready but also not wanting to pre-empt that time. However, for those with more symptomatic illness or who characterised themselves as planners, there was more of a sense of urgency around the need to have advance care planning conversations to ensure that wishes and preferences were stated and recorded at an early opportunity.

*Jenny* [patient’s daughter] *knows where everything is and what’s, what and I’m insured, and they know not to bury me.* **Vera**

Alternatively, others considered there was no best time to have an advance conversation because they did not see the need for one. Sometimes this was because sufficient conversations had occurred within the family or they felt it was not necessary to prepare.

*I don’t need talking through because it’s not that complicated . . . my business, my life, you know . . . it’s quite simple really . . .* **Alan**

### ‘Talking can be tough’

Throughout the interviews, participants described the difficulties they experienced in contemplating the future. They regarded these thoughts as requiring emotional ‘toughness’. For some, this meant not wanting to talk about end of life wishes at all, preferring to maintain a sense of normality by getting on with usual activities

*I mean that’s part of my thinking as well . . . I don’t want to know. I’d rather carry on, you know, doing the garden and playing squash and I went to Centre Parcs [*UK holiday resort*] with my grandchildren . . . and it was just, it was back to normal life . . . and we swam and you know, did things together . . . if you start thinking about the ultimate solution, or the ultimate outcome, then it’s there with you all the time.* **Tom**

Sixteen participants denied having specifically heard the phrase ‘advance care planning’. Where clinicians had apparently initiated these conversations, this had an emotional impact for some. In the context of treatable but not curable cancer, the following illustrative narrative between Anne (patient) and Jane (Anne’s daughter) demonstrates their experiences of shock when planning for end of life was raised unexpectedly

Jane (daughter): *Do you remember when she (registrar - doctor) started asking about what your plans were for the future, if your affairs were in order? Cause, we were really shocked cause the (cancer test) results were really good and everything was stable, wasn’t it?*

Anne (patient): *Yes, I was. I was shocked.*

Jane (daughter): *And a registrar started saying: ‘are all your affairs in order . . . have you got everything prepared for the future’? and we were quite surprised that that was being asked when the results were all stable and there’s nothing to indicate we had to think about it at that time* . . .

Some participants described the existential difficulty of holding contradictory thoughts about living with cancer while simultaneously contemplating end of life preferences. For example, one patient expressed a very firm desire to live whilst also considering the possibility of death

*I don’t see any kind of alternative when you’re talking about advance planning, you know, it’s got to be about end-of-life care . . . that’s what you’re talking about. There’s no other way of describing that. But because I want to live and I’m willing myself to live and I’m determined to live, I find it a struggle to quantify the two . . . that the two can exist side by side even in my head, and I find that difficult.* **Rob**

The advance care planning conversations appeared to be forcing the pace and creating difficulty for Rob despite him feeling that there is no alternative to these conversations.

Many of the participants described a preference for discussing their plans with family members rather than clinicians. Allied to this, for some, was a belief that the family already knew what their wishes were, although this was often related to funeral arrangements rather than preferences for end-of-life care. However, participants were concerned about triggering distressing emotions for their family.

*the problem is with talking with the family is they get upset and then you get upset, so, I mean I’m, I’m talking to you now quite alright but if it were my daughter and she started crying, I’d be upset, you see . . .’*. **Andy**

### Planning in any case

Despite participants not recognising that they had advance care planning conversations or seeing the necessity for discussing their advance wishes and preferences for care with clinicians rather than within families, all except Tom, demonstrated evidence of some planning. Concern for others, especially family members, was frequently expressed and indicates the relational nature of serious illness. Notably, most participants had a ‘do not attempt cardio-pulmonary resuscitation’ order in place or had discussed this, although some interpreted this conversation as implying the likelihood of imminent death (eg. Connie). Conversely Rob, who expressed strongly negative feelings about advance care planning, was nonetheless clear about his thoughts on resuscitation

*I have signed a do not resuscitate form . . . for the NHS and that and I did that with the GP . . . who briefly spoke to me about what that meant, but that’s different to advance care, so I did make the steps . . .* **Rob**

*I was glad it [*advance care planning*] was raised with me but I also felt: ‘you’re asking me because I haven’t got very long . . . you’re not going to leave it another two months and ask me, are you? . . . you’re asking me now because you think it [*death*] might be soon.* **Connie**

The nature of future planning varied according to the circumstances and perspectives of participants. For example, many mentioned making a will, or had addressed lasting power of attorney or had made their funeral arrangements. Participants did not necessarily have fixed ideas but were influenced by their personal circumstances, especially when they had concerns about dependents or strong views on what type of dying they would (not) like to experience. There was a widespread view that aside from medical issues, personal plans are best discussed within the family. For some participants with significant caring responsibilities, advance planning became an imperative that superseded concerns about cancer and dying. For example, Maggie, who was the sole carer for her disabled adult son Dan who would need high levels of support when she died, exemplified this priority.

*I was very aware that time might not be on my side, you don’t know how quickly things are going- so I went into, you know, emergency mode and I mean I got this flat sorted in less than six weeks because I pulled every string going . . . my solicitor knows how focused sort of I am on making sure that things are . . . you know, so I suppose really and truly the responsibility lies with yourself because you’re the only one you know . . . I can’t ask Dan to do everything and sort everything . . .* **Maggie**

In summary, participants lived in a context of overwhelming uncertainty that inhibited the process of advance care planning which they regarded as only applicable once they had clear communication from clinicians that they were near the end of life. They described the existential difficulty of holding contradictory thoughts about living with cancer while simultaneously contemplating end of life preferences. Therefore, most participants did not recognise conversations with clinicians as ‘advance care planning’ even when they apparently discussed (or agreed to) care options like ‘do not resuscitate’ decisions. Most participants preferred to discuss future medical care, social and funeral arrangements with family. However, a few, predominantly those with caring responsibilities such as a disabled son or a partner with dementia, proactively undertook advance care planning, including financial and social arrangements, to protect their dependents.

## Discussion

### Main findings

Our study has gathered the experiences, perceptions and understandings about advance care planning of patients living with treatable but not curable cancer, an increase sub-cohort of cancer patients.^15^ This provides new knowledge that those living with uncertainty of prognosis, rather than those at the end of life, adversely influences their engagement in advance care planning. Across all interviews, there was consistent evidence of poor understanding of the advance care planning conversations and reluctance to engage with clinicians. This may be explained by the dual nature of the status of treatable but not curable cancer which meant that patients had to cognitively hold two divergent beliefs and realities simultaneously.^14^ On the one hand they were offered a range of, often novel, anti-cancer treatments that in some cases meant that they had survived much longer than initially expected, while simultaneously having to come to terms with living with cancer that was not curable and face the reality of dying from the disease. These tensions were revealed in our findings where participants used differing strategies to cope with their cognitive dissonance and perceived uncertainty of illness trajectory. We noted the range of positions adopted by our participants from those demonstrating little acknowledgment of the possibility of dying or avoiding contemplating or discussing it (e.g. Rob, Vera, Mary and Tom) to those who were actively engaging in advance care planning with family, if not with their clinicians (e.g. Maggie and Connie). Others were in a more transitional phase (e.g. Pamela and Peter) either through a sense of not knowing and being unable to plan or currently adjusting to a change in their condition.

### What this study adds?

Advance care planning has been the focus of extensive international research demonstrating that in some contexts it has benefits in cancer care.^1,7,13^ Echoing our findings, there is also evidence that there is a general reluctance by patients to engage in advance care planning with clinicians.^11,20^ Similar hesitancy was reported by the general public.^21^

Our participants appeared to place emphasis on living in the present and lacked a readiness to contemplate future planning. This accords with analysis of transcripts of advance care conservations conducted in an European trial, indicating that a lack of readiness was a barrier.^11,22^ This finding concurs with a qualitative study exploring patient and family experiences of advance care planning which concluded that while such discussions may be appreciated by some patients, many prefer to devote their energies to living well in the moment rather than making plans for an uncertain future especially in relation to therapeutic optimism.^12^

Our findings challenge recommendations that advance care planning should occur as early as possible, with our participants regarding it as only appropriate near end of life. Moreover, they considered palliative care, as applicable only very near the end of life which is not congruent with newer models that emphasise early palliative care that runs concurrently with anti-cancer treatments, even when cancer is incurable.^23–25^

Amongst our cohort of patients, they appeared to discriminate between aspects of advance care planning that are the remit of clinicians compared to their social and personal preferences. We observed that a diagnosis of treatable but incurable cancer with simultaneously being offered more treatment options and increased longevity, presents challenges for patients.^26,27^ Undertaking advance care planning in these circumstances can be difficult for clinicians too.^28^ This occurs against a context of poor understanding of palliative care and a reluctance to talk about death, reported internationally.^12,29,30^

Shared decision making has been advocated as an important aspect of personalised care with the development of innovative tools to facilitate engagement.^31^ However, review evidence indicates that it’s complexity may be a barrier, especially where ambiguity in prognosis is present, as in our cohort of participants.^32^

Similar to other evidence,^13,28,33^ our findings highlight the struggle some of our participants were having in engaging in advance care planning conversations. These recommendations may offer useful clinical applications in developing practice where cancer treatment and palliative care are given concurrently (see Table 3).

**Table 3.** Implications for developing advance care planning practice for patients with treatable but not curable cancer^a^.

Key Findings	Implications for patients	Implications for healthcare providers and organisations
Understandings of illness trajectory (treatable but not curable cancer)		
Participants were uncertain about their illness trajectory and prognosis	Living with uncertainty and needing coping strategies to manage this	Patients need to be supported to manage uncertainty in their illness trajectory
Some participants had lived longer than anticipated which made advance care planning problematic	Planning for the future is difficult in a context of changing prognosis	Communication with patients around the potential variability of life expectancy is essential
The availability of on-going or novel treatment options contributed to uncertainty and was a barrier to advance care planning	Patients may need to factor in the possibility of new treatments when discussing wishes and preferences for care	Helping patients to understand the implications of new treatment options as they arise
Understandings of advance care planning		
Participants often did not recognise having had advance care planning conversations or hearing that phrase	A lack of understanding of the implications of what has been discussed about future care	More clarity required in the language used to describe advance care planning
Most participants thought that discussions about resuscitation preferences should not necessarily be part of advance care planning conversations	Resuscitation preferences are often interpreted as an indication of imminent dying when coupled with advance care planning conversations	Consider how resuscitation and escalating medical treatment decisions are introduced and how they relate to personal/social aspects of advance care planning
Participants were unsure when advance care planning conversations should occur, and preferred to delay them to an undetermined future time when all treatment options had been exhausted	Ambiguity about timing of advance care planning conversation: a recognition that they might be helpful but a reluctance to engage in these conversations	Electronic systems to indicate to healthcare providers when advance care planning might be initiated linked to specific triggers
Preferences for discussing advance care plans		
Many participants preferred to discuss their future plans with family members rather than with health care providers	Patients acknowledge and potentially share their family discussions with their health care providers	Health providers recognise the role that families have in the personal and social aspects of advance care planning
Some participants preferred to take a day-to-day approach rather than plan in the context of uncertainty	Patients exercise choice to manage their illness and live their lives, without necessarily engaging in advance care planning	Health care providers recognise that some patients prefer not to contemplate or plan for the future in the context of uncertainty. Consider the use of tools that allow patients to explore advance care planning at their own pace. Introduce systems and processes that enable flexibility.

a Please note this is not intended to be a checklist.

## Strengths and limitations

This study’s strength lies in using in-depth qualitative interviewing to enable patients to freely express their understandings without *a priori* assumptions about the efficacy of advance care planning. We employed a rigorous qualitative analysis identifying ‘negative cases’ and refining our interpretation with clinicians. Enabling patients to be accompanied by a family member, if desired, could be regarded as a strength, but potentially may have constrained disclosure. Our small sample was recruited from one cancer centre with limited population diversity, where all participants described themselves as White British with Christian or no faith.

## Conclusion

Despite promotion of advance care planning internationally to enable pre-emptive discussion of treatment and end of life care choices earlier in the disease process, our study demonstrates a disparity between these recommendations and views held by patients with treatable but not curable cancer. These findings demonstrate the need to recognise that patients may be reluctant to engage in future planning when living with uncertainty of prognosis. Future research should explore when, or even if, an optimal time exists to initiate such conversations and how best to implement them, and whether introducing these conversations early facilitates better conversations as things change or just results in increased distress.

## Supplemental Material

sj-docx-1-pmj-10.1177_02692163251363752 – Supplemental material for A qualitative study exploring the perceptions and understandings of advance care planning by people with treatable but not curable cancer Supplemental material, sj-docx-1-pmj-10.1177_02692163251363752 for A qualitative study exploring the perceptions and understandings of advance care planning by people with treatable but not curable cancer by Ruth Board, Sean Hughes, Katherine Stewart, Tomoko Lewis and Sheila Payne in Palliative Medicine

sj-docx-2-pmj-10.1177_02692163251363752 – Supplemental material for A qualitative study exploring the perceptions and understandings of advance care planning by people with treatable but not curable cancer Supplemental material, sj-docx-2-pmj-10.1177_02692163251363752 for A qualitative study exploring the perceptions and understandings of advance care planning by people with treatable but not curable cancer by Ruth Board, Sean Hughes, Katherine Stewart, Tomoko Lewis and Sheila Payne in Palliative Medicine
