# Supplementary material for: A qualitative study exploring the perceptions and understandings of advance care planning by people with treatable but not curable cancer
Source: Palliat Med. 2025 Aug 31;39(10):1072–81. doi: 10.1177/02692163251363752 (PMC12640362; doi:10.1177/02692163251363752)
Supplement: sj-docx-1-pmj-10.1177_02692163251363752 – Supplemental material for A qualitative study exploring the perceptions and understandings of advance care planning by people with treatable but not curable cancer [file sj-docx-1-pmj-10.1177_02692163251363752.docx]

**Supplementary file 1: Interview Topic Guide**

**Understanding Oncology Patients Preferences for Advance Care Planning**

**Interview Topic Guide**

**This is an indicative guide to the topics that will be covered in the semi-structured interviews with cancer patients. As is appropriate in qualitative interviews, question order and wording will be tailored to the circumstances of participants and the flow of conversation.**

- **Introduction**
- Introduce the researcher and confirm consent.
- Conversational dialogue to aid developing trust and rapport.
- Completion of demographic characteristics namely: Age, sex, ethnic background, primary cancer type, cancer stage, current treatment

**Topic 1 Exploring understandings of advance care planning in patients receiving palliative systemic therapy for cancer**

- What is your understanding of the term advance care planning?

(If the patient is unaware of the term a brief description will be given).

- What are your experiences so far of advance care planning or planning for the future?
- How would you like your advance care plan be recorded?
- Who should have access to your plan?

**Topic 2: When do patients consider that advance care planning should be introduced**

- How did you feel about the timing of when advance care planning was discussed with you?
- Do you have views about when the best time might be?
- How often do you think advance planning should be discussed?

**Topic 3: Exploring what topics patients would feel comfortable discussing within advance care planning conversations**

- What sort of topics do you think should be discussed during advance care planning?
- What do you think about conversations regarding resuscitation being included in advance care planning?
- How do you feel when advance care planning topics are discussed?
- How does advance care planning change your outlook on the future?

**Topic 4: Patient preferences for who should have these conversations with them**

- Who do you think are the best people or best person to introduce advance care planning to you?
- Who would you like to talk to about advance care planning?
- Do you have views about who should be present during advance care planning conversations?

**Summing up**

- Do you have any further comments you’d like to add.
- Thank the participant for their time and input.
