# Supplementary material for: A qualitative study exploring the perceptions and understandings of advance care planning by people with treatable but not curable cancer
Source: Palliat Med. 2025 Aug 31;39(10):1072–81. doi: 10.1177/02692163251363752 (PMC12640362; doi:10.1177/02692163251363752)
Supplement: sj-docx-2-pmj-10.1177_02692163251363752 – Supplemental material for A qualitative study exploring the perceptions and understandings of advance care planning by people with treatable but not curable cancer [file sj-docx-2-pmj-10.1177_02692163251363752.docx]

**Supplementary file 2: Coding tree**

Oncology Advance Care Planning

Codes

| Name | Description |
| --- | --- |
| 1. Advance care planning conversations | Anything to do with having advance care planning conversations ion the more formal sense: who with, when, what about, the emotional impact together with reasons why these may not be happening and why not. Where should these plans be held, who should have access and how might they be reviewed. |
| Impact of discussing future plans |  |
| No advance care planning conversations |  |
| Reasons for no advance care planning |  |
| I'm not there yet |  |
| Ongoing treatment |  |
| Recording advance care plans |  |
| No written record of wishes |  |
| Who should have access |  |
| Reviewing advance care plans |  |
| Timing of planning conversations |  |
| What could be in an advance care plan |  |
| What is an advance care plan |  |
| Where should an advance care plan be held |  |
| Which professional should discuss advance care planning with you |  |
| Who should be involved in discussing advance care plans |  |
| 2. Do Not Attempt Cardio-Pulmonary Resuscitation | Issues around resuscitation and whether these instructions are in place, or not |
| Do Not Attempt Cardio-Pulmonary Resuscitation conversations |  |
| Do Not Attempt Cardio-Pulmonary Resuscitation equals imminent death |  |
| No Do Not Attempt Cardio-Pulmonary Resuscitation instruction |  |
| 3. Fears and worries | What are participants concerned or worried about. This includes ambivalence and attitudes to ‘knowing’ about what might happen and how the dying process might be. Dilemmas around treatment and whether/what to accept if offered are included here as are worries about leaving family members behind. |
| Ambivalence |  |
| Current symptoms |  |
| Current treatment options |  |
| Don't want to know how long |  |
| Fear of dying process |  |
| Fearing breathlessness |  |
| Fearing pain |  |
| Feeling frightened |  |
| Incurable |  |
| Leaving family behind |  |
| How will they cope |  |
| Professional experience | Some participants had a health care background which enabled comment on systems and organisational issues including perceived deficits in care |
| Shock |  |
| Uncertainty |  |
| What, when, how, where |  |
| 4. Illness and cancer stories | Here, participants recount the experiences of good and bad care for themselves and others (often relatives) which impacts their view on planning for the future |
| Stories of bad previous care |  |
| Stories of good cancer care |  |
| Stories of others illness experiences |  |
| 5. Informal planning work | Participant comments on the more practical and personal aspects of planning that they are engaged with |
| Funeral planning |  |
| Sorting affairs |  |
| Caring responsibilities | Some participants had significant caring responsibilities which are prioritised over concerns about cancer |
| Wishes and preferences |  |
| Not expressed |  |
| Place of dying |  |
| Recording wishes and preferences |  |
| 6. Philosophical approach | Codes to do with the participant approach to their illness and life/death in general |
| Faith and spirituality |  |
| I don't think about it |  |
| I want a normal life |  |
| I'm going to live |  |
| Others worse off than me |  |
| Positive thinking |  |
| Taking it day by day |  |
| Wants honest straight talking |  |
| 7. Sources of support | Where do participants access their support? |
| Cancer centre support |  |
| Family support |  |
| Hospice support |  |
| Primary care support |  |
| Self-reliance |  |
| 8. Talking with... | Participants comment on who they talk to about their illness and plans – or not. |
| Discussions with professionals not needed |  |
| Don't want to talk about it |  |
| Talking with friends |  |
| Talking with the family |  |
| Talking with the General Practitioner or District Nurse |  |
| Talking with the oncologist |  |
| Talking with the palliative nurse specialist |  |
| 9. Timeliness | Issues around timing of conversations in general and when difficult conversations should occur – not necessarily in terms of advance care planning per se. |
